# Chromosome-level genome assembly of *Microplitis manilae* Ashmead, 1904 (Hymenoptera: Braconidae)

**DOI:** 10.1038/s41597-023-02190-3

**Published:** 2023-05-10

**Authors:** Xiaohan Shu, Ruizhong Yuan, Boying Zheng, Zhizhi Wang, Xiqian Ye, Pu Tang, Xuexin Chen

**Affiliations:** 1 Hainan Institute, Zhejiang University, Sanya, 572025 China; 2 Guangdong Laboratory for Lingnan Modern Agriculture, Guangzhou, 510642 China; 3 State Key Lab of Rice Biology, Ministry of Agriculture Key Lab of Molecular Biology of Crop Pathogens and Insects, and Zhejiang Provincial Key Laboratory of Biology of Crop Pathogens and Insects, Zhejiang University, Hangzhou, 310058 China; 4 Institute of Insect Sciences, College of Agriculture and Biotechnology, Zhejiang University, Hangzhou, 310058 China

**Keywords:** Entomology, Genome

## Abstract

*Microplitis manilae* Ashmead (Hymenoptera: Braconidae) is an important parasitoid of agricultural pests in lepidopteran species. So far, two extant genome assembles from the genus *Microplitis* are fragmented. Here, we offered a high-quality genome assembly of *M. manilae* at the chromosome level with high accuracy and contiguity, assembled by ONT long-read, MGI-SEQ short-read, and Hi-C sequencing methods. The final assembled genome size was 282.85 Mb, with 268.17 Mb assigned to 11 pseudochromosomes. The scaffold N50 length was 25.23 Mb, and the complete BUSCO score was 98.61%. The genome contained 152.37 Mb of repetitive elements, representing 53.87% of the total genome size. We predicted 15,689 protein-coding genes, of which 13,580 genes were annotated functionally. Gene family evolution investigations of *M. manilae* revealed 615 expanded and 635 contracted gene families. The high-quality genome of *M. manilae* reported in this paper will be a useful genomic resource for research on parasitoid wasps in the future.

## Background & Summary

*Microplitis manilae* Ahmead (Hymenoptera: Braconidae: Microgastrinae) is a solitary endoparasitoid wasp and is primarily distributed in the Asia-Pacific region^1^. It attacks several lepidopteran species, with *Spodoptera* species being its preferred target, including *S. frugiperda*, *S. exigua* and *S. litura*^2^, which of them are the world’s most significant agricultural pests^3^. *M. manilae* is thought to be an ideal biological control agent for *Spodoptera* spp.

So far, it has approximately 200 species have been recognized within *Microplitis*^1^, and some of them, i.e. *M. croceipes*, *M. demolitor* and *M. mediator*, have been widely used in biological pest control^4–6^. The virulence factors of *Microplitis* wasps that act to suppress or circumvent host immunity are primarily composed of polydnaviruses (PDV), venom, and teratocytes^7,8^. In recent years, the biology, ecology, and interaction with the host of *Microplitis* have been studied^9,10^. The study of the interactions between parasitoid wasps and their host insects, particularly the regulation of host immunity and development by parasitoid wasps, has great potential for increasing the use of parasitoid wasps in sustainable pest management in agriculture. To further understand the complex relationship between parasitoids and their hosts, high quality genome data would play an important role. The genome at the chromosome level may shed light on the evolution of parasites, the mechanisms of parasitism, the potential for developing new techniques for biological control and utilizing natural enemies as resources. However, only two fragmented genomes from the genus *Microplitis* (*M. demolitor* and *M. mediator*) are currently available in NCBI and a chromosome-level genome assembly for *Microplitis* spp. has not been published.

In this study, we used MGI short-read, ONT long-read and Hi-C sequencing technologies to assemble the *M. manilae* chromosome-level genome. The final genome size was 282.85 Mb with a scaffold N50 length of 25.23 Mb, and 268.17 Mb assembled genome sequences were successfully anchored on 11 chromosomes. In total, 15,689 protein-coding genes were identified, and 13,580 of them were functionally annotated.

## Methods

### Insect collection and rearing

The wasps *Microplitis manilae* were collected from maize fields in Dongfang City, Hainan Province, China (18.86°N, 108.72°E) in November 2020 and reared using their host *Spodoptera frugiperda* under laboratory conditions of 26 ± 1 °C, 65 ± 5% RH, and a 14 h light: 10 h dark photoperiod.

### Sequencing

The extraction of DNA and RNA was performed on newly emerged male individuals that had been raised for five or more generations. Genomic DNA was obtained using the Blood & Cell Culture DNA Mini Kit (Qiagen, Hilden, Germany) for both long-read and short-read whole genome sequencing. RNA was isolated using the TRlzol reagent (Vazyme, Nanjing, China). The Hi-C library was generated using the restriction endonuclease DpnII. Long-read sequencing was carried out using the Nanopore PromethION platform (Oxford Nanopore Technologies, UK), with an insert size of approximately 20 kb. Short-read and transcriptome sequencing were performed using libraries with an insert size of 350 bp and sequenced on the MGISEQ. 2000 platform. The total data generated from the long-read sequencing was 76.31 Gb, while the total data generated from the short-read sequencing was 82.60 Gb (Table 1).

**Table 1:** Statistics of the DNA/RNA sequence data used for genome assembly.

Library	Insert size (bp)	Reads number	Raw data (Gb)	N50 read length (bp)	Average coverage (×)
MGI	350	169,141,850	25.37	150	89.70
ONT	20,000	46,509,919	76.31	4,024	269.78
Hi-C	350	279,356,762	41.90	150	148.13
RNA-seq	350	102,189,336	15.33	150	—
Total		597,197,867	158.91		

### Genome size estimation and assembly

The raw reads obtained from the MGISEQ. 2000 platform were subjected to quality control using fastp v0.21.0^11^ to filter adapter sequences and low-quality reads. The remaining reads of MGI library were then used to estimate the genome size of *M. manilae* by GenomeScope v1.0.0^12^ and analyze the 17-mer distribution with Jellyfish v2.3.0^13^. The final genome size was estimated to be 297.29 Mb through K-mer analysis.

**Fig. 1:**
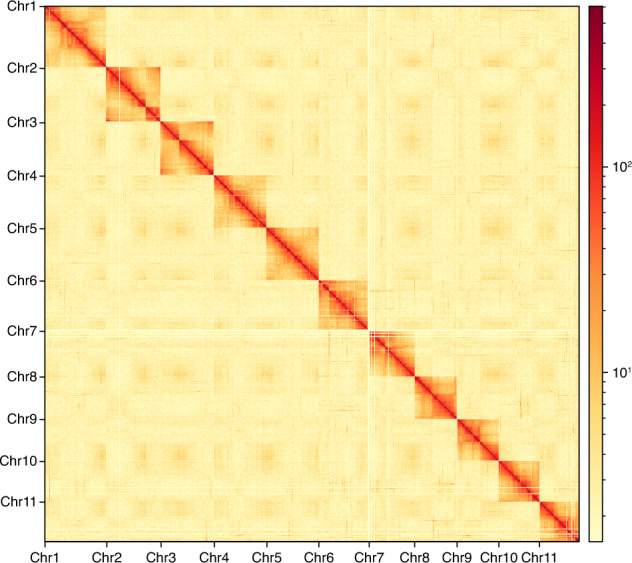
Heat map of Hi-C assembly of *Microplitis manilae*. The scale bar represents the interaction frequency of Hi-C links.

**Fig. 2:**
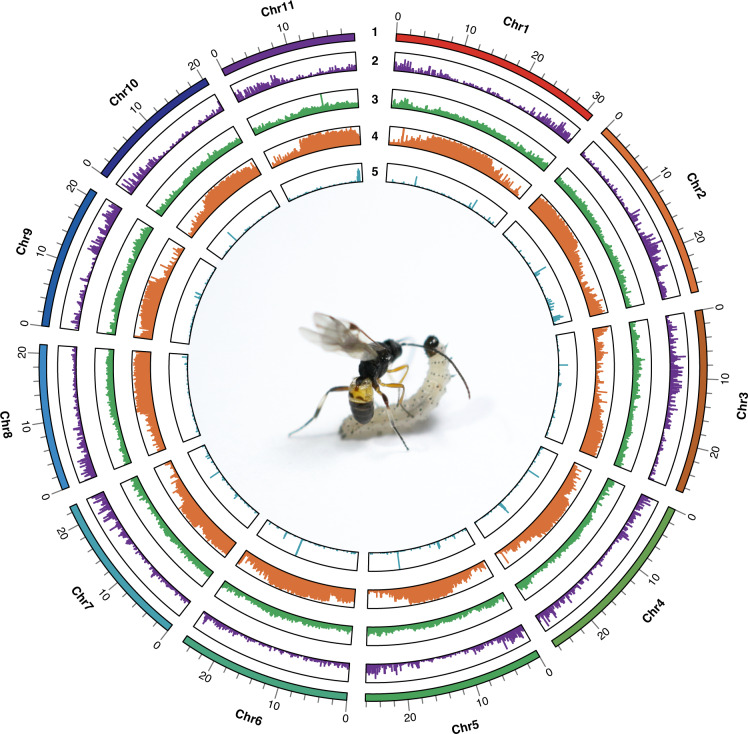
Genome characteristics of *Microplitis manilae*. (1) Pseudo-chromosomes; (2) gene distribution; (3) GC content; (4) repeat distribution; (5) ncRNA distribution.

The draft genome is obtained by first assembling long reads and then polishing the results with short reads, which has been widely used in genome assembly research for different organisms recently^14–18^. NextDenovo v2.5.0 (https://github.com/Nextomics/NextDenovo) was used to assemble the initial assembly with ONT sequences. NextPolish v1.4.0^19^ was then applied to polish the draft genome assembly using MGISEQ sequences. Juicer v1.6.2^20^ was used to align Hi-C reads to the draft assembly and subject them to quality control. 3D-DNA^21^ was used to anchor primary contigs into chromosomes, then corrected the possible errors manually with Juicebox v1.11.08^22^. The final genome assembly of *M. manilae* was 282.85 Mb with a scaffold N50 of 25.23 Mb. The Hi-C analyses scaffolded 11 pseudomolecules (Fig. 1), anchoring 94.81% (268.17 Mb) of the genome assembly of *M. manilae*. The average GC content of *M. manilae* genome assembly was 31.26% (Table 2, Fig. 2).

**Table 2:** Summary statistics of the *Microplitis manilae* genome assembly.

	Statistics
Contig N50 size (bp)	1,792,000
Number of contigs	728
Maximum contig size (bp)	6,940,878
Scaffold N50 size (bp)	25,234,505
Number of scaffolds	363
Maximum scaffold size (bp)	31,007,761
Genome size (bp)	282,852,855
Number of chromosomes	11
Total length of chromosomes (bp)	268,167,060
GC content (%)	31.26

The genome completeness was evaluated with the BUSCO v4.1.4 pipeline^23^, searching against the insect_odb10 database^24^. The analysis identified 98.61% (single-copied genes: 97.88%, duplicated genes: 0.73%), 0.44%, and 0.95% of the 1,367 predicted genes in this genome as complete, fragmented, and missing sequences, respectively. These results suggested the assembled genome is highly complete.

### Genome annotation

The genome of *M. manilae* was annotated for repetitive elements, non-coding RNAs (ncRNAs), and protein-coding genes (PCGs). The Extensive de novo TE Annotator (EDTA) pipeline^25^ was used to build TE libraries for repeat annotation initially. Non-LTR retrotransposons and any unclassified TEs missed by the TE annotators mentioned above were then identified by RepeatModeler v2.0.2^26^. A comprehensive non-redundant TE library was generated combining with above results and Dfam3.2^27^. RepeatMasker v4.1.2 (http://www.repeatmasker.org) was subsequently used to search for known and novel TEs. In the genomic sequences, a total of 152.37 Mb repetitive elements were identified, constituting 53.87% of the total. The most abundant repeating element was DNA transposons (13.54%), followed by long terminal repeats (LTR, 10.43%) and long interspersed nuclear elements (LINEs, 1.75%), while unclassified repeats made up 27.43% of the total (Table 3, Fig. 2). Infernal 1.1.2^28^ was used to identify rRNAs, snRNAs, and miRNAs based on the alignment with the Rfam library^29^. tRNAscan-SE v2.0.6^30^ was used to predict tRNAs. Finally, 1,894 noncoding RNAs were predicted, including 1,269 transfer RNAs (tRNAs), 194 ribosomal RNAs (rRNAs), 74 micro-RNAs (miRNAs), 63 small nuclear RNAs (snRNAs), and 294 others (Table S1, Fig. 2).

**Table 3:** Statistics of repetitive elements in the *Microplitis manilae* genome.

Repeat type	Count	Length occupied (bp)	Proportion in genome	Repeat type	Count	Length occupied (bp)	Proportion in genome
DNA	266	73,824	0.03%	SINE	2	149	0.00%
Academ-1	1	56	0.00%	LINE	163	15,950	0.01%
CMC-Chapaev-3	141	75,850	0.03%	CR1	543	261,360	0.09%
CMC-EnSpm	4,635	781,206	0.28%	Dong-R4	1,202	2,198,887	0.78%
Crypton-I	377	107,590	0.04%	I	142	157,464	0.06%
DTA	13,316	2,972,711	1.05%	I-Jockey	224	199,628	0.07%
DTC	27,206	5,160,763	1.82%	L1	1	88	0.00%
DTH	3,868	452,752	0.16%	L2	1,484	922,420	0.33%
DTM	45,353	7,551,843	2.67%	Penelope	156	61,258	0.02%
DTT	3,317	429,965	0.15%	R1	669	562,557	0.20%
Helitron	26,052	3,328,536	1.18%	R1-LOA	53	99,066	0.04%
MULE-MuDR	276	148,977	0.05%	R2	12	2,043	0.00%
MULE-NOF	1,055	160,661	0.06%	R2-NeSL	41	37,086	0.01%
Maverick	2,851	4,566,223	1.61%	RTE	34	16,849	0.01%
Merlin	341	85,753	0.03%	RTE-BovB	24	1,455	0.00%
PIF-Harbinger	74	11,658	0.00%	RTE-RTE	2	22	0.00%
PIF-Spy	104	66,603	0.02%	RTE-X	289	423,984	0.15%
PiggyBac	281	88,378	0.03%	LTR	193	87,371	0.03%
Sola-2	613	200,965	0.07%	Copia	10,485	2,707,342	0.96%
TcMar-Fot1	707	178,595	0.06%	DIRS	261	272,871	0.10%
TcMar-Mariner	27,955	10,056,986	3.56%	Gypsy	27,718	20,858,254	7.37%
TcMar-Pogo	17	3,525	0.00%	Ngaro	300	45,966	0.02%
TcMar-Tc1	71	15,323	0.01%	Pao	1,442	1,351,002	0.48%
TcMar-Tc4	55	31,151	0.01%	unknown	21,517	4,187,732	1.48%
TcMar-Tigger	7	499	0.00%	MITE			
TcMar-m44	35	29,907	0.01%	DTA	444	49,790	0.02%
Zator	2,950	1,102,391	0.39%	DTC	6,556	724,928	0.26%
hAT	100	47,405	0.02%	DTH	381	38,784	0.01%
hAT-Ac	471	229,325	0.08%	DTM	7,860	687,854	0.24%
hAT-Blackjack	74	85,113	0.03%	DTT	178	16,007	0.01%
hAT-Tag1	5	378	0.00%	RC			
hAT-Tip100	1	125	0.00%	Helitron	5,147	1,690,418	0.60%
hAT-hAT19	602	129,486	0.05%	Satellite	1,728	292,656	0.10%
hAT-hAT5	1	44	0.00%	Simple_repeat	309	28,979	0.01%
hAT-hATm	206	115,356	0.04%	Unknown	271,211	76,081,305	26.90%
				Total	524,155	152,371,448	53.87%

Three different strategies were applied for the annotation of PCGs: transcriptome-based prediction, *de novo* gene prediction, and homology-based prediction. In transcriptome-based prediction, the transcriptome was assembled from RNA-seq alignments by HISAT2 v2.2.1^31^ and the candidate coding region was identified by PASA pipeline v2.4.1 (https://github.com/PASApipeline/PASApipeline). The repeat-masked genome was analyzed using AUGUSTUS v3.3.3^32^ and SNAP v2006-07-28^33^ for *de novo* gene prediction. The protein sequences of hymenopteran species were downloaded from the NCBI Database as references for homology-based prediction. Exonerate v2.4.0^34^ was utilized to align the reference proteins to the genome assembly and predict gene structures. Finally, a consensus gene set was created by integrating the genes predicted by the aforementioned three methods using EVidenceModeler v1.1.1^35^. We predicted 15,689 protein-coding genes for the *M. manilae* genome by combining the evidences from the transcriptome, *ab initio*, and homology-based predictions. The average length of the predicted gene was 8,718 base pairs, while that of a protein-coding region was 1,575 bp. Exon and intron lengths on average were 319 and 1,814 bp, respectively. There were 4.9 exons on average per gene (Table 4).

**Table 4:** Statistics of gene structure annotation in the *Microplitis manilae* genome.

Gene structure annotation	
Number of protein-coding gene	15,689
Mean mrna length (bp)	8,718
Mean CDS length (bp)	1,575
Mean intron length (bp)	1,814
Mean exon length (bp)	319
Mean exons per gene	4.9

Gene functions were annotated using BLASTP v2.9.0^36^ (-evalue 1e-5) to search against UniProtKB (Swiss-Prot + TrEMBL)^37^, and InterProScan 5.52-86.0^38^ to search against the Pfam^39^, CDD^40^, Gene3D^41^, Smart^42^, and Superfamily^43^ databases. The eggnog-mapper v2.1.4^44^ was used to predict conserved sequences and domains, GO terms, and KEGG pathways against the eggnog v5.0 database^45^. A total of 13,580 (86.56%) genes were functionally annotated against the UniProtKB database. In integrating with InterProScan and eggnog annotation results, 13,227 (84.31%) protein-coding genes with protein domains were identified, which were assigned 11,276 COG Functional Categories genes, 9,489 Reactome pathways, 7,819 MetaCyc, 7,722 GO terms, 7,324 KEGG KO terms, and 4,274 KEGG pathways, respectively.

## Data Records

The MGI, ONT, RNA-seq and Hi-C sequencing data used for the genome assembly have been deposited in the NCBI Sequence Read Archive (SRA) database with accession numbers SRR21358828^46^, SRR21358827^47^, SRR21358829^48^ and SRR21358826^49^, respectively, under the BioProject accession number PRJNA872950. The chromosomal assembly has been deposited at GenBank with accession number JAPFQK000000000^50^. Genome annotation information has been deposited in the Figshare database^51^.

## Technical Validation

### Evaluating the quality of the genome assembly

The quality of *M. manilae* genome assembly was evaluated using two approaches. Firstly, sequencing data were mapped to the genome to verify the accuracy, yielding mapping rates of 99.52% for MGI, 94.40% for RNA-seq, and 98.52% for ONT data. Secondly, BUSCO analysis found 98.6% of the 1,367 single-copy orthologues (in the insects_odb10 database) to be complete (97.9% single-copied genes and 0.7% duplicated genes), 0.4% fragmented, and 1.0% missing.

### Chromosome synteny

**Fig. 3:**
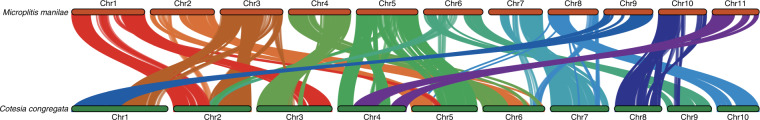
Chromosomal synteny between *Microplitis manilae* and *Cotesia congregata* genomes.

Chromosome synteny between *M. manilae* and *Cotesia congregata* was detected by MCScanX^52^ with default parameters. The genome assembly of *C. congregata*^53^ was retrieved from NCBI with accession number GCA_905319865.3. The visual diagram was generated using TBtools^54^. The synteny of the *M. manilae* assembly was compared to that of *C. congregata*, a closely related species of the subfamily Microgastrinae. The results showed a low level of synteny between *M. manilae* and *C. congregata* (Fig. 3). A number of fusion and fission events were detected between these two wasps. For instance, Chr11 and a part of Chr5 of *M. manilae* were syntenic to Chr4 of *C. congregata*, whereas Chr1 of *M. manilae* was syntenic to a portion of Chr2 and Chr3 of *C. congregata*. Low genome synteny was also identified between *Nasonia vitripennis* and *Pteromalus puparum*, both of which are members of the same family Pteromalidae^55^.

### Gene annotation validation

**Fig. 4:**
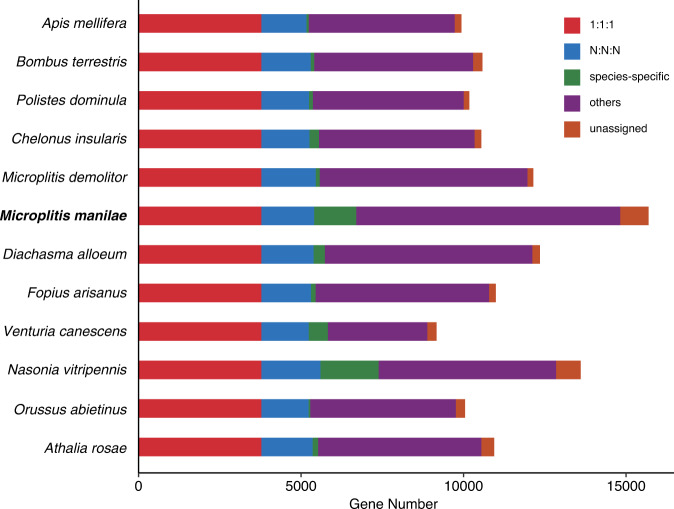
Distribution of genes in different Hymenoptera species. “1:1:1” represents shared single-copy genes, “N:N:N” as multicopy genes shared by all species, “others” as unclassified orthologs, “unassigned” as orthologs which cannot be assigned into any gene families (orthogroups).

OrthoFinder v2.5.4^56^ was utilized to infer sequence orthology, based on protein annotation sequences of 11 additional hymenopteran organisms retrieved from NCBI, including *Apis mellifera*, *Athalia rosae*, *Bombus terrestris*, *Chelonus insularis*, *Diachasma alloeum*, *Fopius arisanus*, *M. demolitor*, *Nasonia vitripennis*, *Orussus abietinus*, *Polistes dominula*, and *Venturia canescens* (Table S2). A total of 132,122 genes were assigned to 12,544 gene families. Among them, 4,910 gene families were presented in all the species genomes, with 3,780 single-copy and 1,130 multicopy gene families. In the 15,689 predicted genes of *M. manilae*, 14,822 (94.47%) were grouped into 9,725 families. There were 1,295 genes in 241 families unique to *M. manilae* (Fig. 4, Table S3).

**Fig. 5:**
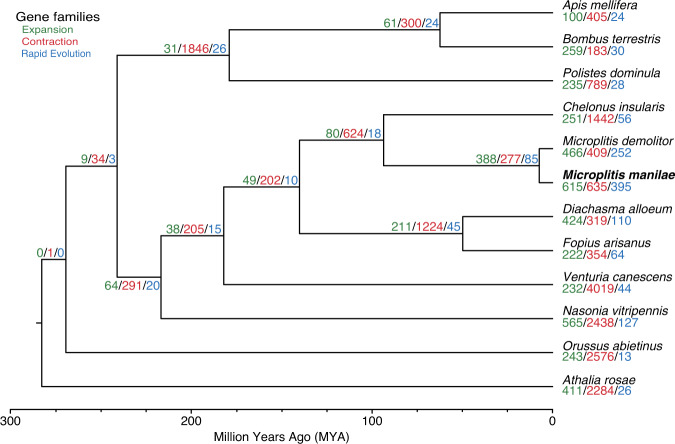
Phylogenetic and gene family evolution analyses of *Microplitis manilae* and 11 other Hymenoptera species. The bootstrap values of all nodes are supported at 100/100. Node values indicate the number of gene families showing expansion, contraction, and rapid evolution. The scale at the bottom of the figure represents the divergence time.

All single-copy protein sequences were concatenated into one data matrix after being aligned with MAFFT v7.427^57^. The phylogenetic tree was constructed using IQ-TREE v2.0.5^58^ with the best model (JTT + F + R7) estimated by ModelFinder^59^. Statistical support for the phylogenetic trees was evaluated by Ultrafast bootstrap^60^ analysis using 1000 replicates. The phylogenetic tree reconstructed by IQ-TREE had high bootstrap support values. The topology of the phylogeny was consistent with that of the previous study^61^. The MCMCTree package in PAML v4.9j^62^ was used to estimate divergence times. Based on a previous study, five calibration time points were used: root holometabolous: <300 million years ago (mya); Orussoidea + Apocrita: 211–289 mya; Apocrita: 203–276 mya; Aculeata: 160–224 mya; and Ichneumonoidea: 151–218 mya^61^. As expected, our analysis revealed that *M. manilae* was closely related to *M. demolitor* and these two species diverged approximately 7.6 mya (Fig. 5). CAFE v4.2.1^63^ was used to estimate gene family expansions and contractions with a *p* value of 0.01. Finally, we found 615 and 635 gene families experienced expansions and contractions in *M. manilae*, respectively, and 395 (310 expanded and 85 contracted) of them were rapidly evolved (Fig. 5).

## Supplementary information

Supplementary table

## Data Availability

This work did not utilize a custom script. Data processing was carried out using the protocols and manuals of the relevant bioinformatics software.
